# Waterborne Disease Outbreak Detection: A Simulation-Based Study

**DOI:** 10.3390/ijerph15071505

**Published:** 2018-07-17

**Authors:** Damien Mouly, Sarah Goria, Michael Mounié, Pascal Beaudeau, Catherine Galey, Anne Gallay, Christian Ducrot, Yann Le Strat

**Affiliations:** 1 Santé Publique France, the French National Public Health Agency, 94 410 Saint-Maurice, France; sarah.goria@santepubliquefrance.fr (S.G.); pascal.beaudeau@santepubliquefrance.fr (P.B.); catherine.galey@santepubliquefrance.fr (C.G.); anne.gallay@santepubliquefrance.fr (A.G.); yann.lestrat@santepubliquefrance.fr (Y.L.S.); 2 Unité D’évaluation Médico-Economique, Université Paul Sabatier, CHU 31059 Toulouse, France; mounie.michael.12@gmail.com; 3 Institut National de la Recherche Agronomique, UR346-Unité d’Épidémiologie Animale, 63 122 Saint Genès Champanelle, France; christian.ducrot@inra.fr

**Keywords:** waterborne disease outbreak, simulation study, health insurance data, space–time detection

## Abstract

Waterborne disease outbreaks (WBDOs) remain a public health issue in developed countries, but to date the surveillance of WBDOs in France, mainly based on the voluntary reporting of clusters of acute gastrointestinal infections (AGIs) by general practitioners to health authorities, is characterized by low sensitivity. In this context, a detection algorithm using health insurance data and based on a space–time method was developed to improve WBDO detection. The objective of the present simulation-based study was to evaluate the performance of this algorithm for WBDO detection using health insurance data. The daily baseline counts of acute gastrointestinal infections were simulated. Two thousand simulated WBDO signals were then superimposed on the baseline data. Sensitivity (Se) and positive predictive value (PPV) were both used to evaluate the detection algorithm. Multivariate regression was also performed to identify the factors associated with WBDO detection. Almost three-quarters of the simulated WBDOs were detected (Se = 73.0%). More than 9 out of 10 detected signals corresponded to a WBDO (PPV = 90.5%). The probability of detecting a WBDO increased with the outbreak size. These results underline the value of using the detection algorithm for the implementation of a national surveillance system for WBDOs in France.

## 1. Introduction

Outbreaks of infectious waterborne diseases are still a public health concern in developed countries [1,2]. Most of the time, acute gastrointestinal infections (AGIs) are the syndrome involved. In most of the waterborne disease outbreaks (WBDOs) reported in the last decade in Europe, the United States of America, and Canada, several hundred to several thousand people became ill after drinking water contaminated by infectious pathogenic agents. In rare cases, tens of thousands of people were affected. This occurred for example in two waterborne cryptosporidiosis outbreaks which occurred in 2010 and 2011 in Sweden, infecting 27,000 and 20,000 people, respectively [3,4], and in the 1993 disaster in Milwaukee, which affected 400,000 people [5]. There is also concern in France with respect to WBDOs [6], but to date, in the absence of a specific nationwide surveillance system, the detection of these events is mainly based on the voluntary reporting of clusters of AGIs by general practitioners to health authorities. The mean outbreak size of reported WBDOs (ranging from several hundred to thousands of AGI cases) suggests that only the most important events are reported. Despite the need for the development of a specific nationwide surveillance system to improve the detection of outbreaks caused by contaminated drinking water in France, and accordingly help health authorities with the microbial risk management of drinking water, the creation of such a specific surveillance system is a challenge for French authorities.

For several years, *Santé publique France* (the French Public Health Agency) has been using the French Health Insurance Administrative Database (*Système national d’information inter régimes de l’Assurance maladie*, SNIIRAM) for the syndromic surveillance of the medicalized AGI cases by employing a specifically developed algorithm [7]. Medicalized AGI cases are aggregated by day and by zip code. Although not all medicalized AGI cases can be specifically attributed to drinking water contamination, the correspondence between AGI cases and water distribution zones (DZs) at a local level provides the opportunity to study the ecological relationship between tap water and infectious gastrointestinal diseases. Medicalized AGI data have already proven relevant for studying the relationship between tap water quality parameters (e.g., turbidity) and the incidence of AGIs [8,9], and also for retrospectively identifying and describing outbreaks of AGIs notified by general practitioners [8,10]. Consequently, using these data to develop a specific automated nationwide system for local WBDO detection offers a promising way forward.

By so doing, an integrated approach to detect and localize WBDOs using medicalized AGI cases from SNIIRAM data was published in 2017 by Coly et al. [11]. The authors’ approach relied on a space–time statistical method developed by Kulldorff [12] used to detect local outbreaks of AGIs. Their approach integrates the DZ as the ecological unit of exposure to tap water. A detected outbreak of AGIs localized in a DZ (compared with no outbreak in the surrounding DZs) is considered as a potential WBDO and is characterized by epidemiological criteria (day one of detected signal, duration, number of expected cases, and number of observed cases, DZ identity code). Each outbreak is retrospectively investigated in terms of various environmental criteria during the days before the onset of the outbreak: weather (e.g., heavy rain) and technical incidents in drinking water treatment (e.g., chlorination breakdown, alarm malfunction) or in the distribution system (e.g., water pipe breaks). However, the performance of this integrated approach in the implementation of nationwide retrospective automated WBDO detection has not been evaluated to date. Another detection method, inspired by field investigator practices in France and based on the comparison of the incidence ratio mean between the zip code and specific French administrative districts (called *départements*) was published in 2016 [13]. However we did not decide to select this method for evaluation as its theoretical foundations were considered insufficient [14].

A simulation-based study was performed to evaluate Coly et al.’s detection algorithm in order to implement a nationwide surveillance system. Simulation studies were performed to evaluate surveillance methods and disease control measures [15,16]. To our knowledge, to date, no simulation-based study has been specifically developed to evaluate an automated WBDO detection system. One of the major challenges regarding outbreak detection systems of contaminated water is to identify the largest number of clusters corresponding to real WBDO (i.e., maximizing the sensitivity) while avoiding clusters that are inconsistent with WBDO assumption (i.e., minimizing the number of false positives).

The objective of our study was therefore to evaluate, through simulations, the performance of the integrated approach developed by Coly et al. for WBDO detection [11] and to highlight the DZ and outbreak features which most influence WBDO detection.

## 2. Materials and Methods

### 2.1. Study Area and Period

Two French *départements*, Puy-de-Dôme and Isère, with 655,498 and 1,253,410 inhabitants, respectively [17], were selected. Puy-de-Dôme was included in the previous study by Coly et al. for the construction of the WBDO detection algorithm. Isère is known for chronic microbiological pollution of DZ. The period studied extended from 1 January 2010 to 31 December 2013.

### 2.2. Reference Health Data

Health data represented medicalized AGI cases from the French National Health Insurance Information System (*Système national d’information inter régimes de l’Assurance maladie,* SNIIRAM). SNIIRAM aims to evaluate beneficiaries’ healthcare consumption and associated expenditures. It covers more than 98% of the French population and records all patient reimbursements for out-of-pocket medical procedures, medications, and payments to professionals for consultations [18]. Almost all medicalized AGI cases in France, irrespective of the route of infection (contaminated drinking water, person-to-person transmission, food poisoning), can be identified from SNIIRAM using a specifically developed algorithm detailed elsewhere [7]. Using the algorithm, medicalized AGI cases were selected from people who consulted a general practitioner and went to a pharmacy to buy medications prescribed to treat AGI. To be included, the individual had also to meet a set of conditions regarding the delay of purchase after the visit to doctor, and the combination of drugs prescribed. A national survey study showed that 33% of individuals with symptomatic AGI consulted a doctor in France [19]. Cases were aggregated by day and residence zip code at the municipality level (note: *départements* are comprised of smaller municipal areas).

### 2.3. Simulation Study

**Figure 1:**
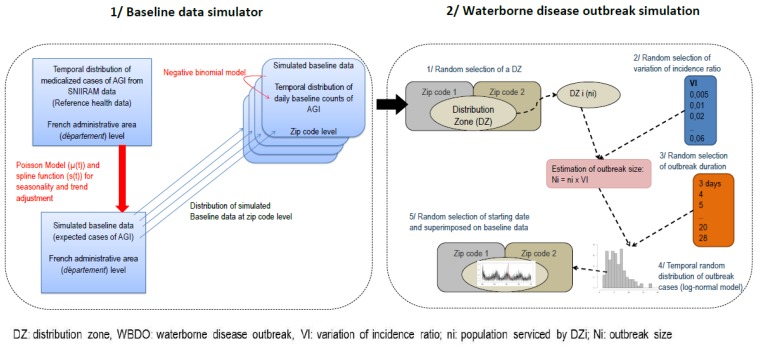
Algorithm of the overall process for simulation of baseline data and WBDOs. SNIRAM: *Système national d’information inter régimes de l’Assurance maladie* (the French Health Insurance Administrative Database); AGI: acute gastrointestinal infection.

Several steps were implemented to simulate WBDOs (Figure 1).

We simulated the daily baseline counts of AGI at the zip code level using the SNIIRAM data. A variety of simulated WBDO signals were then superimposed on the baseline data. The simulation study was based on a methodology developed to evaluate the performance of an algorithm for outbreak detection of infectious diseases, which met the objectives of our study [15].

### 2.4. Simulation of Baseline Data

Baseline counts of AGI were first generated at the *département* level (1) and then at the zip code level (2).

**Figure 2:**
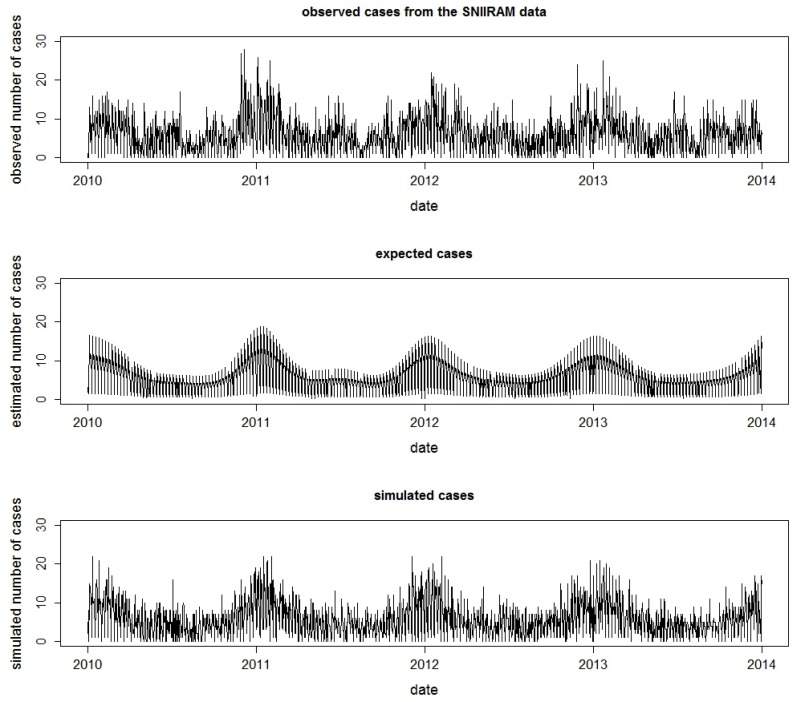
Simulation of time series of incident AGI cases before the inclusion of the simulated WBDO: daily number of observed AGI cases from the French Health Insurance Administrative Database (SNIIRAM) between 1 January 2010 and 31 December 2013 (*n* = 8677) (**top**), number of estimated expected cases (**middle**), and number of simulated cases (**bottom**) for a zip code with 18,541 inhabitants.

(1) A Poisson regression was used to model the daily observed counts of AGI at the *département* level (SNIIRAM data). A thin-plate regression spline [20] was used to model trend and seasonality in order to account for the seasonality of the AGI, in particular the variability of winter viral pandemic of AGI. Adjustments were made for days of the week and holidays [21].
(2) The estimated expected values obtained from the regression model at (1) were then distributed at the zip code level in proportion to the number of cases observed in the SNIIRAM data. Finally, to introduce stochasticity, daily counts of AGI cases were simulated using a negative binomial distribution [15,21] (Figure 2).

### 2.5. The Simulation Process of Waterborne Disease Outbreaks

The spatial unit of interest for the WBDO simulation in our study was the DZ. By definition, a DZ delivers water of homogenous quality to consumers, meaning that all people serviced by the same DZ are exposed to the same risk in terms of water quality apart from situations of backflows, and where contamination directly enters the network. There are 25,000 DZs and 35,000 zip codes in France. As the health outcome (i.e., AGI cases) was simulated at the zip code level, when the selected DZ serviced more than one zip code, AGI outbreak daily cases were distributed according to the proportion of inhabitants serviced by the DZ in each zip code [22].

**Figure 3:**
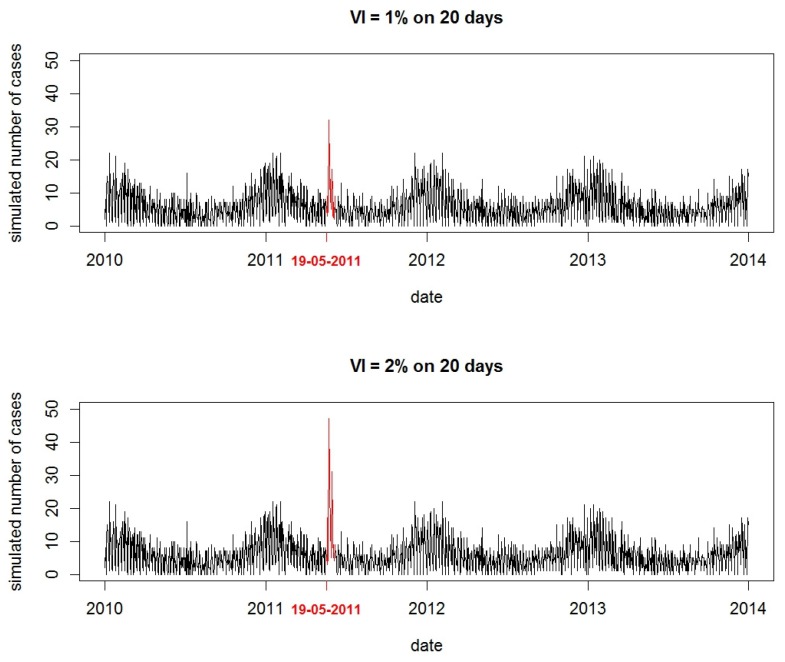
Illustration of two simulated outbreaks starting on 22 September 2011 in a zip code of 18,541 inhabitants serviced by only one DZ, with a variation of incidence ratio (VI) of 1% (**top**) and 2% (**bottom**), and with a 20-day duration.

1. DZs were randomly selected. DZs servicing fewer than 200 inhabitants were excluded from the simulation study to ensure statistical power of detection and because of their reduced impact on public health.
2. For each simulation, the variation of incidence ratio (VI), defined as the proportion between the number of outbreak AGI cases and the number of expected cases of AGI (baseline data) during the outbreak period, was randomly selected between 0.5% and 6%. These values were chosen according to what we observed in previous WBDOs [10].
3. The outbreak duration was randomly selected between 3 and 28 days in accordance with the observed values in reported WBDOs [6].
4. The outbreak size, that is, the number of AGI cases in the outbreak, was generated by multiplying the VI by the number of inhabitants serviced by the DZ.
5. Finally, outbreak cases were distributed over time according to a log-normal distribution [15,21] (Figure 3). The parameters of the log-normal distribution used to shape the time distribution of the outbreak AGI cases were randomly chosen between 0.33 and 0.5 for the median, and fixed at 0.5 for the standard deviation [10,21]. When the selected DZ serviced more than one zip code, daily cases in the AGI outbreak were then distributed according to the proportion of inhabitants serviced by the DZ in each zip code.

A total of 2000 simulations were run (1000 for each of the two French *départements* studied). Each simulated set included the simulation of the baseline data and a WBDO.

The simulation study was performed using the R software version 3.3.0 (R foundation for Statistical Computing, Vienna, Austria).

### 2.6. Detection of Simulated Waterborne Disease Outbreaks

The WBDO detection method used is detailed elsewhere [11]. First, an algorithm was used for grouping zip codes (and corresponding AGI cases) which share the same DZ so that tap water exposure could be taken into account in the detection process. Then the space–time permutation scan statistic developed by Kulldorff et al. [12] was applied to grouped zip codes. The scan statistic was based on overlapping cylinders to define a scanning window. In our study, the scanning window is represented by grouping zip codes sharing the same DZ and defined by the algorithm.

With a space–time permutation scan statistic, expected cases are calculated using observed cases. A generalized likelihood ratio is then used as a measure of the evidence that a tested cylinder contains an outbreak (i.e., number of observed cases exceeds the number you would typically expect to see in a comparable period of time). The cylinder with the maximum generalized likelihood ratio constitutes the space–time cluster of cases least likely to occur by chance, and consequently it is the primary candidate for a true outbreak. Within the space–time permutation model, adjustments were made for days of the week and holidays.

This detection study was performed using SaTScan version 9.3 [23] and R version 3.3.0.

### 2.7. Data Analysis

#### 2.7.1. Evaluation Method

The running of the scan on the simulated dataset generated a set of clusters. All clusters associated with a statistical threshold (*p*-value) of 0.05 were considered, whether they revealed a true alarm (i.e., WBDO detected) or not. A true alarm was declared if at least one detected day and one detected zip code corresponded to the days and zip codes involved in the simulated WBDOs [15]. The other clusters were considered as false alarms.

To evaluate the performance of the WBDO detection method we considered the sensitivity (Se) and the positive predictive value (PPV). Sensitivity was estimated as the ratio between the true alarm and the number of simulated WBDOs (i.e., 1000 WBDOs generated per administrative area). The PPV was defined as the ratio between the number of true alarm and the number of all the clusters detected associated with a statistical threshold of 0.05. The Se and VPP were described by administrative area, by DZ size (inhabitants served), by outbreak size, and by season.

#### 2.7.2. Factors Associated with WBDO Detection

A multivariate Poisson regression was performed on true alarms to identify the factors associated with WBDO detection and to estimate the strength of these associations [21]. Five dependent variables were considered: outbreak duration, size of the DZ population, outbreak incidence ratio, outbreak size, and season (“winter” for December, January, February and March/“other seasons” for April to November). We tested for potential interactions among these factors. Incidence rate ratios (IRRs) and their 95% confidence intervals (CIs) were computed. The IRR is the ratio between the incidence rate in a considered group and the incidence rate in the reference group. All analyses were performed using Stata 12.0 (StataCorp LP, College Station, TX, USA).

## 3. Results

### 3.1. Description of Simulated WBDO

Simulated WBDOs involved between 1 and 7392 AGI outbreak cases (median = 22; mean = 96). Most (90%) included 200 AGI cases or fewer (Table 1). The mean outbreak duration was 15 days (3 to 28 days). All DZ sizes were represented: 35.8% of the randomly selected DZs serviced 200 to 500 people, 21.9% between 500 and 1000 people, 15.5% between 1000 and 2000 people, 21.1% between 2000 and 10,000, and 5.9% more than 10,000 people. Among all the simulated WBDOs, 26.7% (*n* = 534/2000) involved a DZ which serviced more than one zip code: 162 WBDOs generated for Isere with 2 to 13 zip codes were serviced by the same DZ, and 372 WBDOs for Puy-de-Dôme with 2 to 53 zip codes were serviced by the same DZ.

### 3.2. Sensitivity and Positive Predictive Value of the Detection Method

**Figure 4:**
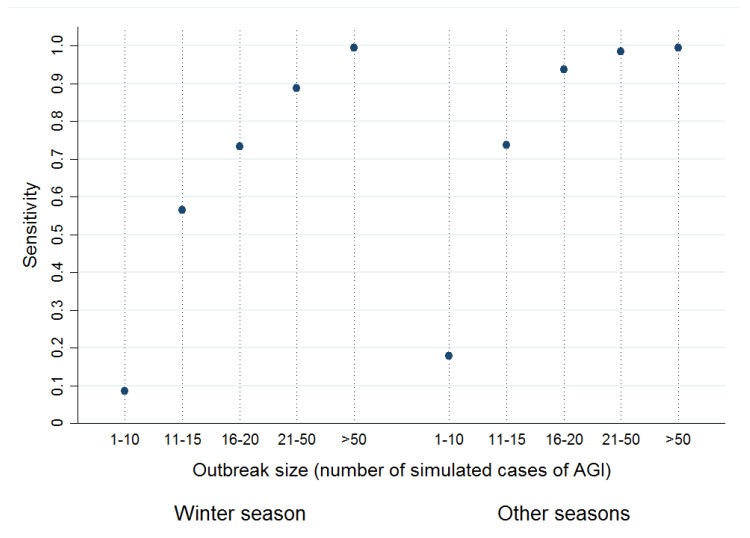
Sensitivity of detection method according to outbreak size (number of simulated AGI cases) and season (winter: December, January, February, March).

Almost three-quarters of the 2000 simulated WBDOs were detected (sensitivity = 73.0%). More than 9 out of 10 detected signals corresponded to a simulated WBDO (PPV = 90.5%). Sensitivity increased with DZ size and with outbreak size (Table 2). Moreover, WBDOs in non-winter seasons (hereafter “other seasons”) were better detected than WBDO simulated during the winter season. Indeed, to reach the same sensitivity value of 75%, WBDO size had to be greater in the winter season than in other seasons (at least 15 cases versus 10 cases) (Figure 4).

For WBDOs occurring in more than one zip code (a total of 534 for both departments studied), the sensitivity of detection was higher than for WBDO associated with a DZ servicing only one zip code (78.3% and 71.1%, respectively), while the PPV was stable (90.2% versus 91.1%, respectively). For half of the WBDOs associated with a DZ servicing several zip codes, 80% of these zip codes were included in the detected signal for Isere and 50% for Puy-de-Dôme.

The undetected WBDOs involved mostly small DZ (200–500 inhabitants) and few outbreak cases (Table 1).

### 3.3. Factors Associated with WBDO Detection

In the multivariate Poisson regression, the outbreak size, the VI, and the duration and the season of WBDOs were all significantly associated with detection (Table 3). The interaction of VI and the outbreak size was significant. WBDOs involving at least 10 AGI cases, with a 14-day duration or less, and occurring between April and November, had a higher probability of being detected. The variable “outbreak size” had the strongest association with detection.

## 4. Discussion

### 4.1. Simulation Process

The first step of this simulation-based study was to generate the baseline incidence of the disease. This step employed a published method [15] adapted for AGI epidemiology by adding a flexible adjustment function (spline) to account for high winter incidence of AGIs due to the enteric virus outbreak. Days of the week and holidays were adjusted for to reflect the closure of pharmacies during weekends and holidays. This ensured an acceptable representativeness of seasonality and of AGI incidence (Figure 1).

Simulated WBDOs were generated using a log-normal distribution model [15]. The parameters used to build these epidemic signals were inspired by past outbreaks of waterborne AGIs. Accordingly, the chosen epidemic duration ranging (from 3 to 28 days) and the chosen VI (between 0.5% and 6%) are realistic. When compared with the health impact of WBDOs assessed in cohort studies in which the attack rate varied between 30% and 50%, the AGI outbreak cases observed in SNIIRAM data are less frequent. This difference is probably due to several factors, including healthcare-seeking behaviors: in France, the mean consultation rate for AGI is quite low at 32% [19] and depends on age and pathogen agent. The true distribution of cases during outbreaks is described in a previous article which compared, for two WBDOs, the true distribution of cases identified in cohort studies among the impacted population and the true distribution of medicalized cases identified in the SNIIRAM database [10]. Results highlighted a good temporal correlation between both data sources. Moreover, a descriptive study of 11 WBDOs reported in France between 1998 and 2006 gives the main parameters of outbreak distributions [6].

One limitation of this study was the choice not to perform WBDO simulations for DZ servicing fewer than 200 inhabitants. This prevented us from being able to evaluate the algorithm for these DZs. However the public health concern is less important for these small zip codes.

Another limitation regards the use of the SNIIRAM database. Disease severity associated with an epidemic may be a criterion that influences control measures. Nevertheless, medicalized AGI cases (i.e., those who consulted a doctor and subsequently went to the pharmacy with a prescription) identified from the health insurance database cannot be distinguished in terms of illness severity in the absence of a specific association between severity and the drugs prescribed. This said, medicalized AGI cases represented almost 32% of AGI cases in one French national survey [19]. In that study, less than 1% of all individuals with an AGI went to a hospital for consultation. The main reported reasons for consulting were: prolonged symptoms (49%), vomiting (31%), diarrhea (28%), and unusual symptoms (27%). The main reasons for not consulting were: quick recovery/no serious symptoms (64%) and feeling that a consultation was not necessary (47%). After multivariate analysis, gender, age, duration of illness, and symptoms (headache) were associated with consultation for AGIs. Given these results, AGI medicalized cases can be considered as the most severe cases.

### 4.2. Algorithm Performance for WBDO Detection

Globally, the algorithm has a high sensitivity (detecting 73% of simulated WBDOs) and a high positive predictive value among the detected signals (90.5% corresponded to simulated WBDOs). These indicators reached, respectively, 99.2% and 86.7%, for DZs servicing more than 10,000 people (Table 2). The performance (sensitivity and positive predictive value) of the algorithm mainly depended on the serviced population size and the outbreak size. The influence of the season and the number of zip codes served by the same DZ were less important (Table 2 and Table 3). If we focus on the influence of the serviced population size, a threshold of 500 inhabitants resulted in increased sensitivity, from 50% (fewer than 500 people served) to more than 75% (more than 500 people served) of detected WBDO. Likewise, when the outbreak size exceeded 10 AGI cases, the sensitivity was four times greater than with smaller outbreaks (10 cases or fewer). For these two parameters, the most significant variations in sensitivity were observed when between 200 and 2000 people were serviced (from 49.4% to 94.1%, respectively) and between fewer than 10 AGI cases to over 50 (17.2% and 99.3%). Nevertheless, the 10-case sensitivity threshold should be treated with caution and is not the main consideration for the implementation of surveillance system of WBDO. With respect to the positive predictive value, there is no outbreak size threshold which could influence detection. Therefore, the criterion for considering large epidemics or large DZ is a criterion of public health efficiency (avoided cost from an avoided case).

### 4.3. Factors Influencing Detection

In addition to the evaluation of the performance of the detection algorithm, the simulation study also allowed us to identify and quantify the three factors which most influence the performance of WBDO detection as follows: outbreak size, duration and season (“winter” or “other seasons”). The existence of a significant interaction between the outbreak size and the variation of incidence led us to consider the results according to three classifications of the incidence ratio (0.5% to 2%; 2% to 4%; 4% to 6%) (Table 3).

From the results of our analysis, outbreak size had the strongest association with detection sensitivity, especially for a variation of incidence value below 4%. Above this value, outbreak size was no longer associated with detection. For variation of incidence rate between 0.5% and 2%, the outbreak size has a dominant effect (vis-à-vis duration and season), with a detection capacity 13 times greater for an outbreak of 20 AGI cases or more than for an outbreak of 10 or fewer cases (the incidence rate ratio is 7.7 when going from fewer than 10 cases to 15 cases). For a variation of incidence between 2% and 6%, the detection ratio (IRR) did not exceed 3 between the most extreme values (more than 50 cases versus fewer than 10 cases). These results suggest a strong improvement in detection ability for WBDOs with more than 10 AGI cases and a variation of incidence greater than 2%. These values are consistent with the previous study’s results which described the detection algorithm and its application to real health data [11]. Of the 11 clusters detected in this study, the values of the medication rate in the population (indicator close to the variation of incidence) ranged from 0.7% to 4.8%, and the cluster size from 21 to 67 AGI cases.

As mentioned above, “duration” and “season” also affected detection but much less substantially than outbreak size. WBDOs with a lower variation of incidence (0.5–2%) were primarily affected by these three factors. Accordingly, the number of detected WBDO was 1.3 times higher in non-winter season outbreaks of AGI. Similarly, outbreaks which lasted less than 14 days were better detected than longer outbreaks.

### 4.4. International Comparison

To our knowledge, few previously published simulation studies on WBDO detection exist. Different Canadian research studies have presented an agent-based simulation model for generating realistic multivariable outbreak signals [24]. This model was used to simulate a WBDO caused by *Cryptosporidium*, taking into account parameters for population, water consumption, and disease progression. To verify whether the simulation model produced credible results, the authors attempted to replicate the largest documented WBDO of cryptosporidiosis, which occurred in Milwaukee in 1993. During that outbreak, over 400,000 people were estimated to have diarrhea attributable to acute *Cryptosporidium* infection [5]. The results showed that the simulated curve was slightly more positively skewed and peaked one to two days earlier than the historically observed curves. These simulated data were then used to improve early outbreak detection using a hidden Markov model [25].

Although French health insurance data constitute an adequate source for the retrospective surveillance of WBDO, they do not allow—at least for the moment—the possibility of implementing a prospective approach within the context of a public health alert system.

## 5. Conclusions

Our study presents a global approach for simulating AGI baseline data using reference health data and superimposing simulated WBDOs. The algorithm for WBDO detection was evaluated as being able to detect almost 90% of WBDOs, with few false positive alarms. We also estimated the factors which most influence WBDO detection. The results of our study underline the value of using the detection algorithm for the implementation of a national surveillance system for WBDOs in France upon which to base public health action.

## Figures and Tables

**Table 1:** Description of simulated WBDOs by *département*.

Variables	*n*	Both *départments*					Puy-de-Dôme					Isère				
		Total	Detected	%	Undetected	%	Total	Detected	%	Undetected	%	Total	Detected	%	Undetected	%
		2000	1460		540		1000	726		274		1000	734		266	
DZ size (number of inhabitants served by DZ)																
	200–500	715	353	49.4	362	50.6	385	201	52.2	184	47.8	330	152	46.1	178	53.9
	501–1000	437	330	75.5	107	24.5	204	153	75.0	51	25.0	233	177	76.0	56	24.0
	1001–2000	309	264	85.4	45	14.6	128	107	83.6	21	16.4	181	157	86.7	24	13.3
	200–10,000	421	396	94.1	25	5.9	188	171	91.0	17	9.0	233	225	96.6	8	3.4
	>10,000	118	117	99.2	1	0.8	95	94	98.9	1	1.1	23	23	100.0	0	0.0
Outbreak size (number of simulated cases of AGI)																
	Min	1	5		1		2	6		2		1	5		1	
	*p*10	5	11		2		5	11		2		5	12		2	
	Median	22	38		6		22	35		6		23	39		6	
	Mean	96.2	128.8		8.1		122.5	165.3		8.9		69.9	92.6		7.3	
	*p*90	199	271		14		255	412		15		140	187		14	
	Max	7392	7392		133		5551	5551		133		7392	7392		33	
Duration (days)																
	Min	3	3		3		3	3		3		3	3		3	
	Median	16	15		17		15	14		16		16	15		18	
	Mean	15.4	15.0		16.4		15.2	14.8		16.3		15.6	15.2		16.5	
	Max	28	28		28		28	28		28		28	28		28	
DZ area (number of municipalities served)																
	1	1466	1042	71.1	424	28.9	628	445	70.9	183	29.1	838	597	71.2	241	28.8
	>1	534	418	78.3	116	21.7	372	281	75.5	91	24.5	162	137	84.6	25	15.4
Season																
	Winter	605	414	68.4	191	31.6	298	199	66.8	99	33.2	307	215	70.0	92	30.0
	Other seasons	1395	1046	75.0	349	25.0	702	527	75.1	175	24.9	693	519	74.9	174	25.1

**Table 2:** Sensitivity and predictive positive value of the detection method according to outbreak size, distribution zone (DZ) size, season, and DZ area.

Variables	Total					Isère				Puy-de-Dôme			
		Se		PPV		Se		PPV		Se		PPV	
		%	*N*1	%	*N*2	%	*N*1	%	*N*2	%	*N*1	%	*N*2
		73.0	2000	90.5	1614	73.4	1000	89.0	825	72.6	1000	92.0	789
DZ size (number of inhabitants served by DZ)													
	200–500	49.3	715	88.0	401	46.0	330	82.1	185	52.2	385	93.0	216
	501–1000	75.5	437	91.4	361	75.9	233	92.6	191	75.0	204	90.0	170
	1001–2000	85.4	309	92.9	284	86.7	181	91.2	172	83.5	128	95.5	112
	2001–10,000	94.0	421	91.4	433	96.5	233	89.2	252	90.9	188	94.4	181
	>10,000	99.1	118	86.6	135	100.0	23	92.0	25	98.9	95	85.4	110
Outbreak size (number of simulated cases)													
	1–10	15.2	466	77.1	92	13.8	224	77.5	40	16.5	242	76.9	52
	11–15	68.5	312	91.4	234	64.6	150	85.8	113	72.2	162	96.6	121
	16–20	86.4	170	91.8	160	83.3	90	90.3	83	90.0	80	93.5	77
	21–50	95.3	449	90.8	471	97.9	240	89.0	264	92.3	209	93.2	207
	>50	99.5	603	91.3	657	100.0	296	91.0	325	99.0	307	91.5	332
Season													
	Winter *	68.4	605	87.7	472	70.0	307	84.3	255	66.7	298	91.7	217
	Other season	74.9	1395	91.5	1142	74.8	693	91.0	570	75.0	702	92.1	572
DZ area (number of municipalities served)													
	1	71.0	1466	90.2	1155	71.2	838	88.7	673	70.8	628	92.3	482
	>1	78.2	534	91.0	459	84.5	162	90.1	152	75.5	372	91.5	307

Se: sensitivity; PPV: positive predictive value; *N*1: number of WBDO simulated; *N*2: number of clusters detected with *p*-value ≤ 0.05; DZ: distribution zone; * Winter: December, January, February, March.

**Table 3:** Final multivariate regression model with factors significantly associated with WBDO detection, stratified by variation of the incidence ratio.

Variables		VI: 0.5%–2.0%			VI: 2.0%–4.0%			VI: 4.0%–6.0%		
		*n* = 642	IRR	[95% CI]	*n* = 659	IRR	[95% CI]	*n* = 699	IRR	[95% CI]
Outbreak size (number of simulated cases)										
	1–10	331	ref		129	ref		6	ref	
	11–15	79	7.70	[5.03–11.74]	130	2.01	[1.55–2.61]	103	1.59	[0.71–3.58]
	16–20	40	10.30	[6.79–15.60]	51	2.62	[2.02–3.39]	79	1.91	[0.86–4.28]
	21–50	96	12.80	[8.7118.82]	151	2.85	[2.24–3.62]	202	1.96	[0.88–4.37]
	>50	96	13.70	[9.29–20.07]	198	2.92	[2.30–3.70]	309	2.03	[0.91–4.53]
Season										
	Winter *	193	ref		193	ref		219	ref	
	Other seasons	449	1.37	[1.20–1.56]	466	1.11	[1.03–1.19]	480	1.05	[1.01–1.10]
Outbreak duration (days)										
	3–7	131	ref		136	ref		133	ref	
	8–14	173	0.84	[0.73–0.97]	180	1.00	[0.92–1.09]	184	0.97	[0.94–1.01]
	15–21	178	0.77	[0.66–0.90]	170	0.89	[0.81–0.97]	178	0.94	[0.90–0.99]
	22–28	160	0.64	[0.54–0.76]	173	0.89	[0.81–0.98]	204	0.93	[0.89–0.97]

VI: variation of the incidence ratio; IRR: incidence rate ratio; CI: confidence Interval; WBDO: waterborne disease outbreak; * winter: December, January, February, March.
